# Supplementary material for: Gene Cloning, Tissue Expression Profiles and Antiviral Activities of Interferon-β from Two Chinese Miniature Pig Breeds
Source: Vet Sci. 2022 Apr 15;9(4):190. doi: 10.3390/vetsci9040190 (PMC9030596; doi:10.3390/vetsci9040190)
Supplement: Supplementary file 1 [file vetsci-09-00190-s001.zip › Sublementary Table 1.pdf]

Supplementary Table S1. GenBank accession numbers of different animal species used for sequence alignment.

Species	GenBank Accession no
Bama minature pig	OL446997
Banna miniature pig	OL446998
Sus scrofa	AEQ59453
Bos Taurus	XP_002689587
Ovis aries	XP_004004449
Capra hircus	AFU82528
Equus caballus	NP_001092910
Felis catus	NP_001009297
Canis lupus familiaris	NP_001129259
Camelus bactrianus	XP_010944318
Homo sapiens	NP_002167
