# Supplementary material for: Gene Cloning, Tissue Expression Profiles and Antiviral Activities of Interferon-β from Two Chinese Miniature Pig Breeds
Source: Vet Sci. 2022 Apr 15;9(4):190. doi: 10.3390/vetsci9040190 (PMC9030596; doi:10.3390/vetsci9040190)
Supplement: Supplementary file 1 [file vetsci-09-00190-s001.zip › Supplementary Figure 1.pdf]

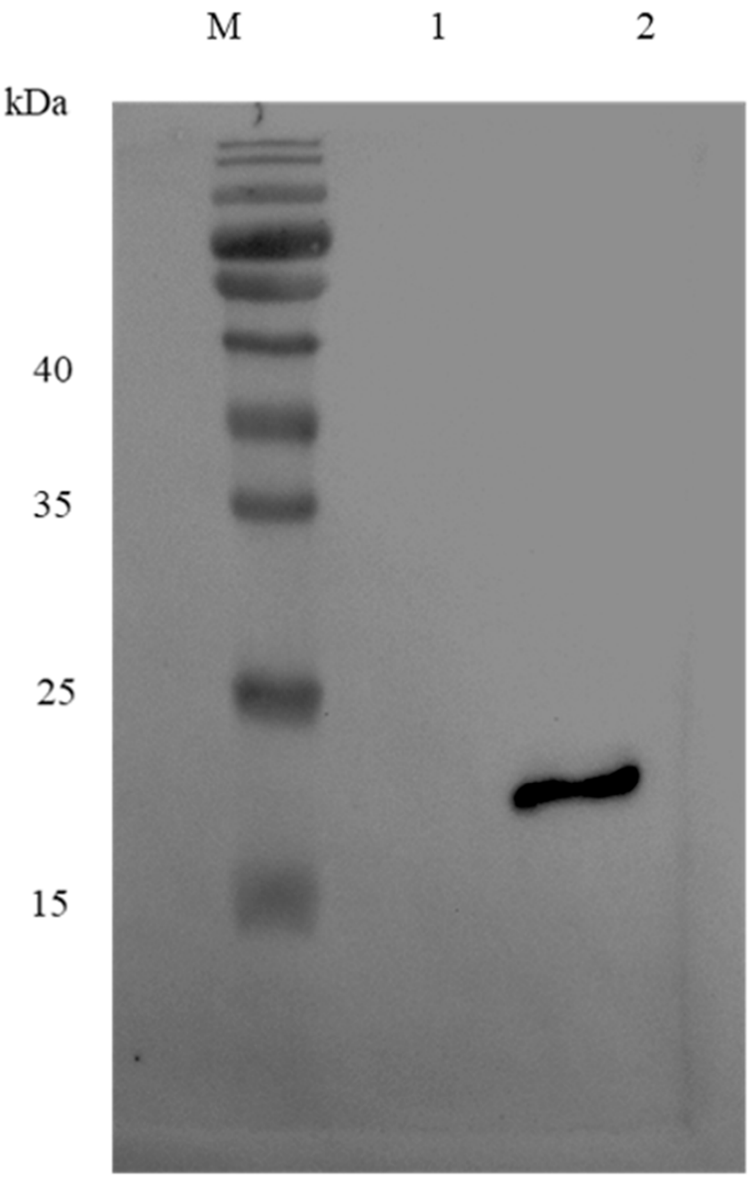

Supplementary Figure S1. Western blotting, Identification of rPoIFN-β from Bama miniature pig: (1) Induced pet30a; (2)purified rPoIFN-β
